# Strategic Approach to the Globalization of Sasang Constitutional Medicine

**DOI:** 10.1093/ecam/nep202

**Published:** 2011-06-08

**Authors:** Myeong Soo Lee

**Affiliations:** Korea Institute of Oriental Medicine, Daejeon 305-811, Republic of Korea

## Abstract

The workshop on “Strategic Approach to the Globalization of Sasang Constitutional Medicine (SCM)” was held in the Korea Institute of Oriental Medicine (KIOM) on September 18, 2009. This workshop was designed to discuss and brainstorm the strategic approach to the globalization of SCM, one of the unique systems in Korean Traditional Medicine, with three topics and an extensive panel discussion. Professor Edwin Cooper, Editor-in-Chief of *eCAM*, gave a commemorative lecture for publication of the second supplement of *eCAM* entitled “Sasang Constitutional Medicine as a Holistic Tailored Medicine”. The other two presenters suggested some practical methods for globalization of SCM on the basis of their experiences. After the three main presentations, there was a panel discussion session for further development of workshop topics, extended by five other external experts. They discussed the benefits, limitations and essentials for globalization of Korean Traditional Medicine, specifically SCM, from bench to bedside.

One special issue, which we called the Korea Institute of Oriental Medicine (KIOM) supplement, was published in early September by *eCAM* entitled “Sasang Constitutional Medicine (SCM) as a Holistic Tailored Medicine" [1]. This issue consisted of one editorial, a commentary, six reviews and three original articles on basic science and three clinical analyses for SCM. Editorial for this supplement opened the issue on the unique contribution of SCM to complementary and alternative medicine (CAM) [2]. A brilliant commentary briefs the possible application of systems biology for the interpretation of SCM [3]. Among six reviews, the most fundamental one was the hypothesis that SCM shares the same vision as holistically tailored medicine [4]. The other five reviewers included a psychological profile of SCM [5], perspectives of the human body and SCM [6], current research on the methods of diagnosing SCM [7], the interpretation of SCM using a genetic approach [8] and current clinical evidence regarding SCM acupuncture [9]. The three original articles of basic science were feature selections on the use of facial image for distinguishing SC [10], the association between genetic polymorphism of multidrug resistance 1 genes and SC [11] and the effect of herbal remedies on the basis of SCM for obesity-related genes in mice [12]. The additional three original articles of clinical analysis included the relationship between skin humidity and SC [13], quantitative SC diagnosis methods [14] and relationship between SC and risk factor of diabetes mellitus [15].

The publication of this special issue led to conduct aworkshop on “Strategic Approach to the Globalization of Sasang Constitutional Medicine". This was held in the KIOM, the unique government-funded institute for Korean Traditional Medicine in Korea, on September 18, 2009, in Deajeon, Korea supported by the Division of Constitutional Medicine, KIOM (Principal investigator: Dr. Jong Yeol Kim). This workshop was designed to discuss and brainstorm the globalization of SCM, one of the unique systems in Korean Traditional Medicine, with three presentations and a panel discussion.

Professor Edwin L. Cooper, the Editor-in-Chief of *eCAM*, gave a commemorative lecture on publication of the second supplement of *eCAM*. He made a brief introduction to this supplement and gave a 1-hour lecture about how to globalize SCM. He showed the print version of supplement with beautiful cover of “Banquet of Seowangmo, Joseon dynasty (the name of Kingdom in Korea from 1392 to 1910)", which was reproduced with the kind permission of the Los Angeles County Museum of Art. He introduced the content of this supplement briefly and introduced SCM as a unique contribution to the growing field of CAM. According to Prof. Cooper, the supplement to *eCAM* represents a milestone, in that it is the first publication in this area of alternative medical practices. He also described the rigorous peer-review process for the supplement and summarized how SCM represents a potentially fruitful modern approach to an ancient practice that will probably attract more and more attention. In conclusion, he finished his talk with the promise of that this application of an ancient system to international human health would be forthcoming.

Dr. Myeong Soo Lee of KIOM presented practical strategies for the publication of CAM researches in Science Citation Index journals. His main topic and contents were drawn from his personal experience of submission, publication and peer review of other scientist's papers. His talk started with the main factors for successful publication and the procedures for submission and the peer-review process. In particular, he showed several submission platforms of online journals and gave many personal tips for successful publication.

Professor Han Chae from Pusan National University focused on how to enhance the publication of Sasang typology based on personal experiences in Korea and the United States. He also discussed how the uniqueness of SCM made publishing articles on the subject really tricky. He highlighted the most common errors that authors make in writing for publication, for example, trying to explain the whole subject in one manuscript, without giving enough reliable references supporting the underlying notions. His issue was the research model and operational definition that should be built up with sufficient references.

After the three main presentations, there was a panel discussion session for further development of workshop topics, extended by five other external experts: Profs Young E. Earm (Seoul National University), Byung Hee Koh (Kyung Hee University), Heon M. Lim (National Research Foundation of Korea), Kwang-Sup Soh (Seoul National University) and Jong Yeol Kim (KIOM). They discussed the benefits, limitations and essentials for globalization of Korean Traditional Medicine, specifically SCM, from bench to bedside.

The next day, I hosted a meeting of the Editorial Board in Seoul. The attendees were Profs Edwin L. Cooper (Editor-in-Chief), IL-Moo Chang (Kyung Hee University), Byung Hee Koh (Kyung Hee University), Sanghoon Lee (Kyung Hee University) and Myeong Soo Lee (KIOM), with Prof. and Dean, Seung-Hoon Choi (Kyung Hee University) as observer. We discussed several topics, including the second quarter's reports from *eCAM* and the future role of editorial members for *eCAM*.
